# Ultrafast electron diffraction from transiently aligned asymmetric top molecules: Rotational dynamics and structure retrieval

**DOI:** 10.1063/4.0000163

**Published:** 2022-10-17

**Authors:** Kyle J. Wilkin, Yanwei Xiong, Haoran Zhao, Sri Bhavya Muvva, Sajib Kumar Saha, Martin Centurion

**Affiliations:** Department of Physics and Astronomy, University of Nebraska-Lincoln, Lincoln, Nebraska 68588, USA

## Abstract

Ultrafast electron diffraction (UED) from aligned molecules in the gas phase has successfully retrieved structures of both linear and symmetric top molecules. Alignment of asymmetric tops has been recorded with UED but no structural information was retrieved. We present here the extraction of two-dimensional structural information from simple transformations of experimental diffraction patterns of aligned molecules as a proof-of-principle for the recovery of the full structure. We align 4-fluorobenzotrifluoride with a linearly polarized laser and show that we can distinguish between atomic pairs with equal distances that are parallel and perpendicular to the aligned axis. We additionally show with numerical simulations that by cooling the molecules to a rotational temperature of 1 K, more distances and angles can be resolved through direct transformations.

## I. INTRODUCTION

Gas-phase electron diffraction has been used, to great success, in determining the structure of gaseous molecules to high precision.^1,2^ Around the turn of the century, the works of Weber and Zewail added a new component, ultrafast temporal precision, allowing changes in the structure on the order of a few picoseconds to be extracted.^3–5^ Temporal resolution in ultrafast electron diffraction (UED) has continued to advance over the last 20 years through the addition of techniques such as radio frequency (RF) compression of the electron bunches,^6–8^ and acceleration to MeV energies using RF cavities.^9^ These methods have allowed extraction of excited state dynamics on timescales approaching a hundred femtoseconds.^10–15^

While the advancements in temporal resolution have been impressive, UED signals are usually relegated to one spatial dimension, extracting changes in bond lengths. Information in higher dimensions can be retrieved through comparison to quantum calculations. Bond angles,^16^ the isolated dynamics of two excited states following traversal of a conical intersection,^11^ vibrational motion in a radical,^13^ and full excited state structures^17^ have all been retrieved through the use of comparison with simulated structures and trajectories. As interesting and important as these studies have been, it is an imperative part of the scientific process to be able to retrieve the structures from experimental data before comparing to theoretical simulations.

Extracting the higher dimensional information without comparison to theory requires additional methods, such as manipulations of the molecular ensemble. One such method that has proven successful using UED is laser-induced molecular alignment.^18,19^ Such alignment has successfully been combined with UED to retrieve the structure of both linear^20^ and symmetric top molecules.^21^ As the majority of molecules do not fall into either of these categories, the next logical step would be to apply these techniques to asymmetric tops. Unfortunately, the three unique rotational constants corresponding to the principal axes of an asymmetric top molecule make alignment more difficult experimentally and more difficult to model theoretically. Diffraction techniques using both electrons and x rays have shown that transient alignment of asymmetric tops can be recorded,^22,23^ although no diffraction studies to date have succeeded in extracting multi-dimension real space information. It has been shown theoretically that the structure can be retrieved from UED of aligned asymmetric top molecules using an iterative algorithm,^24^ although this has not yet been demonstrated experimentally.

**FIG. 1.**
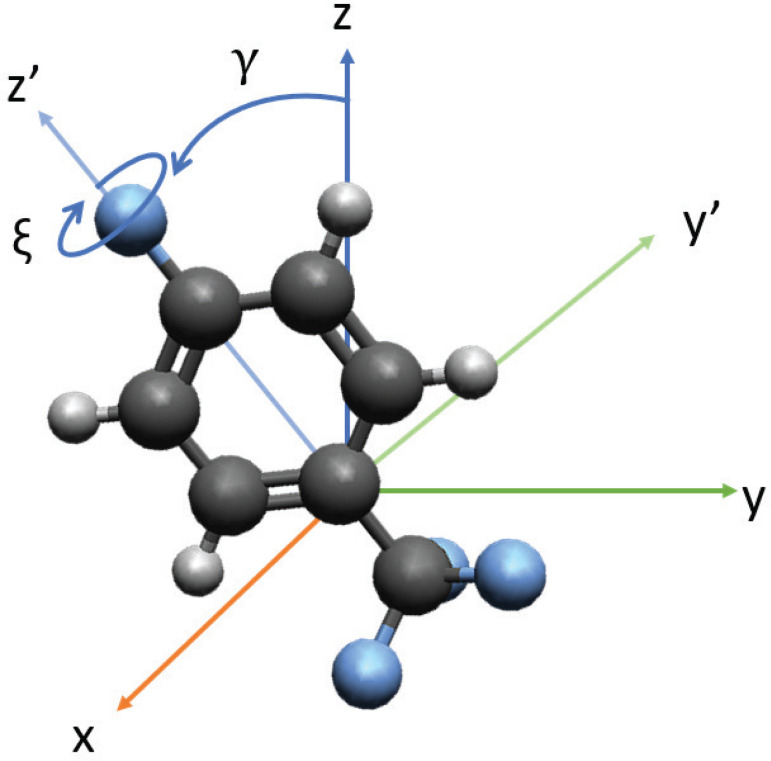
Description of the orientation of the two most polarizable axes with respect to the laser polarization axis, *z*. The angle of the most polarizable axis, *z*′, is given by *γ*, and the angle of rotation of the second most polarizable axis, *y*′, is given by *ξ*. Note: Conventional naming of the angles is not used due to conflict with conventional designation of the scattering angle, θ.

Here, we present proof-of-principle experimental and numerical UED results from impulsively aligned asymmetric top 4-fluorobenzotrifluoride (FC_6_H_4_CF_3_) (Fig. 1). The diffraction patterns show good agreement with theoretical predictions up to a momentum transfer of 11 Å^−1^, an improvement over previous UED experiments on aligned molecules of a factor of two.^22^ We then transform the diffraction images to real-space using a two-dimensional Fourier transform followed by an Abel inversion (2DFT-A), again showing excellent agreement with simulations. The separation of distances parallel and perpendicular to the molecular axis represents the first experimental evidence of two-dimensional (2D) structural information of an asymmetric top directly from diffraction data.

Furthermore, we show with numerical simulations that with improved alignment more angles can be resolved within the transformed diffraction data, leading to a significant increase in structural information. An initial temperature of 1 K leads to accurate retrieval of many distances and angles in the molecule using the direct 2DFT-A transform. This temperature is experimentally feasible, although the low-density presents a challenge of UED experiments and would require a higher electron beam current than currently feasible with our instrument.

## II. THEORY

In this section, we briefly describe the general background and equations used in gas phase UED pump probe experiments. We then describe how diffraction simulations from any arbitrary angular distribution are produced. Finally, we describe how, with given initial conditions, time-dependent angular distributions of a molecular ensemble are calculated.

### A. Electron diffraction and real-space transformations

Electron diffraction from gaseous molecules has been studied extensively and is briefly described here. A sample of molecules in a gas are probed by a pulsed electron beam with a known energy. The diffraction intensity for elastic scattering recorded by a given pixel on the detector is given by

$$I\left(\theta ,\vartheta \right)=I\left(\vec{s}\right)=\frac{I_{0}}{D^{2}(s(\theta ))}\sum_{i=1}^{N}\sum_{j=1}^{N}f_{i}(s(\theta ))f_{j}^{*}(s(\theta ))e^{i\vec{s}\left(\theta ,\vartheta \right)\cdot {\vec{r}}_{ij}},$$ (1)

where θ and ϑ are the polar and azimuthal diffraction angles, respectively, $I_{0}$ is a constant related to the brightness of the electron beam, D is the distance from the interaction point to the diffraction ring associated with θ, $\vec{s}$ is the vector given by the momentum change, with $s=\frac{4\pi }{\lambda }\text{sin}(\frac{\theta }{2})$, and λ is the de Broglie wavelength of the electrons, ${\vec{r}}_{ij}$ is the interatomic distance vector between the ith and jth atoms, and $f_{i}$ is the form factor for the ith atom.

The total intensity can be separated into two parts.

$$I\left(\vec{s}\right)=\frac{I_{0}}{D^{2}(s)}\sum_{i=1}^{N}{\left|f_{i}\left(s\right)\right|}^{2}+\frac{I_{0}}{D^{2}(s)}\sum_{i=1}^{N}\sum_{j=1\neq i}^{N}f_{i}{\left(s\right)f}_{j}^{*}\left(s\right)e^{i\vec{s}\cdot {\vec{r}}_{ij}}.$$ (2)

The first term contains no structural information, only information pertaining to the atoms of the molecule, and is referred to as atomic scattering, $I_{at}$. The second term, containing the ${\vec{r}}_{ij}$ term, contains all the interatomic distances of the molecule and is dubbed molecular scattering, $I_{\mathrm{mol}}$. When the molecules are randomly oriented, integration over the full volume of orientation yields

$$I\left(s\right)=\frac{I_{0}}{D^{2}(\theta )}\sum_{i=1}^{N}{\left|f_{i}\right|}^{2}+\frac{I_{0}}{D^{2}(\theta )}\sum_{i=1}^{N}\sum_{j=1\neq i}^{N}f_{i}f_{j}^{*}\frac{\text{sin}(sr)}{sr}.$$ (3)

It should be noted that total scattering for randomly oriented molecules is a function of the magnitude of *s* only, making the diffraction azimuthally symmetric on the detector. This corresponds to the experimental diffraction patterns before the laser interacts with the sample and is referred to as static diffraction throughout this work.

Taking the static diffraction as a reference, we can isolate the time-dependent signal through the diffraction difference method,^16^

$$\begin{array}{c} \Delta I\left(\vec{s},t\right)=I\left(\vec{s},t\right)-I\left(s,-\infty \right)\\ =I_{\mathrm{mol}}\left(\vec{s},t\right)+I_{at}\left(s,t\right)-I_{\mathrm{mol}}\left(s,-\infty \right)-I_{at}\left(s,-\infty \right)=I_{\mathrm{mol}}\left(\vec{s},t\right)-I_{\mathrm{mol}}\left(s,-\infty \right)={\Delta I}_{\mathrm{mol}}. \end{array}$$ (4)

Since $I_{at}$ is constant throughout the dynamics, it simply cancels through the diffraction difference. Taking the difference helps to remove noise and artifacts in the images.

The amplitude of $I_{\mathrm{mol}}$ drops off quickly as a function of scattering angle and requires normalization. A common practice in UED is to divide the molecular scattering by $I_{at}$ and multiply by *s*, giving the modified scattering intensity, *sM*. This removes the dampening of the diffraction signal, allowing the entire range of the diffraction signal to be viewed simultaneously.

$$sM\left(\vec{s},t\right)=\frac{I_{\mathrm{mol}}\left(\vec{s},t\right)}{I_{at}\left(s\right)}\cdot s.$$ (5)

The time-dependent *sM* follows directly from replacing $I_{\mathrm{mol}}$ in Eq. (5) with either ΔI or ${\Delta I}_{\mathrm{mol}}$,

$$\Delta sM\left(\vec{s},t\right)=\frac{\Delta I\left(\vec{s},t\right)}{I_{at}\left(s\right)}\cdot s=\frac{{\Delta I}_{\mathrm{mol}}\left(\vec{s},t\right)}{I_{at}\left(s\right)}\cdot s.$$ (6)

For the case of randomly oriented molecules, a sine transformation of the sM gives the pair distribution function (PDF).

$$\mathrm{PDF}\left(r\right)=\int_{0}^{s_{\text{max}}}sM\;\text{sin}(sr)e^{-ds^{2}}ds,$$ (7)

where $s_{\text{max}}$ is the maximum scattering vector recovered by the detector. A dampening term, $e^{-ds^{2}}$, is included to reduce the edge effects caused by $s_{\text{max}}$, where d is a constant.

For the case of diffraction from aligned molecules where the scattering intensity depends on two variables, the real space information can be obtained by first Fourier transforming the images in both components of the momentum transfer vector perpendicular to the direction of the incident electron beam (*s_y_* and *s_z_*), then applying an Abel inversion.^15,25^ This gives the modified pair distribution function (MPDF) as described in Ref. 15, which is the cylindrical projection of the three-dimensional (3D) PDF of the molecule convolved with the angular distribution.

$$\mathrm{MPDF}\left(r,\alpha \right)={\mathrm{Abel}}^{-1}F^{-2D}\left\{I\left(\vec{s}\right)\right\}=\sum_{i=1}^{N}\sum_{j=1\neq i}^{N}g_{ij}(\alpha )\frac{\delta ({r-r}_{ij})}{r_{ij}^{2}}⊗F\left\{f_{i}f_{j}^{*}\right\},$$ (8)

where $g_{ij}(\alpha )$ is the angular distribution of the atom pair *ij*, and α is the angle between ${\vec{r}}_{ij}$ and the laser polarization axis. The relation of the atom-pair angular distribution $g_{ij}(\alpha )$ and probability density of the molecular orientation is given by (1) in Ref. 26. $\frac{\delta ({r-r}_{ij})}{r_{ij}^{2}}$ gives peaks for atomic distances $r_{ij}$, $F\left\{f_{i}f_{j}^{*}\right\}$ represents the Fourier transform of the scattering form factors, and A⊗B represents the convolution of A and B.

The transform of $\Delta I\left(\vec{s},t\right)$ gives the time-dependent Δ*MPDF*,

$$\Delta \mathrm{MPDF}\left(r,\alpha ,t\right)={\mathrm{Abel}}^{-1}F^{-2D}\left\{\Delta I\left(\vec{s},t\right)\right\}.$$ (9)

### B. Alignment of a molecular ensemble with a linearly polarized laser pulse

The interaction between a linearly polarized laser pulse and an asymmetric top molecule induces excited rotational states, essentially providing an angular “kick” to the molecule. For the case of 4-fluorobenzotrifluoride, the principal moments of inertia and the polarizable axes of the molecule overlap. We take the most polarizable axis (MPA), and the principal axis, to point along *z*′, as seen in Fig. 1. The interaction causes the MPA to align along the polarization axis of the laser pulse after some time, with the time dependent on the rotational constants and polarizability of the molecule and the intensity of the laser pulse.

We use the Euler angles to define the rotation of the molecule. We take *γ* to be the angle between *z*′ and *z*, which corresponds to the angle between the MPA and the laser polarization. The value of ⟨cos^2^(*γ*)⟩ for a molecular ensemble gives a measure of the alignment of the MPA, with random alignment ⟨cos^2^(*γ*)⟩ = 0.33, perfect alignment (all the molecules have MPA parallel to the laser polarization axis) ⟨cos^2^(*γ*)⟩ = 1, partial alignment (molecules have the MPA pointing closer to the laser polarization axis) 0.33 < ⟨cos^2^(*γ*)⟩ <1, and partial anti-alignment (molecules have the MPA pointing along the x–y plane) 0 ≤ ⟨cos^2^(*γ*)⟩ < 0.33.

The second most polarizable axis (SMPA) is given by *y*′ in Fig. 1. The interaction also causes rotation of the SMPA described by *ξ*, the angle of rotation about *z*′. The value of ⟨cos^2^(*ξ*)⟩ does not describe the relationship between the SMPA and the laser polarization axis. Consider a 4-fluorobenzotrifluoride molecule with the benzene ring in the *z*–*y* plane, then ⟨cos^2^(*ξ*)⟩ gives the relationship of *y*′ and *y*_0_′, where *y*_0_′ is *y* rotated by angle *γ* for a given molecule. A random (uniform) distribution gives ⟨cos^2^(*ξ*)⟩ = 0.5, perfect alignment (all molecules in the ensemble have their SMPA parallel to *y*_0_′) ⟨cos^2^(*ξ*)⟩ = 1, partial alignment (molecules have their SMPA pointing preferentially in the *y*_0_′ direction) gives 0.5 < ⟨cos^2^(*ξ*)⟩ < 1, partial anti-alignment (molecules have their SMPA pointing preferentially perpendicular to the *y*_0_′ direction) gives 0 ≤ ⟨cos^2^(*ξ*)⟩ < 0.5.

The distribution of the molecules along the azimuthal angle describing the rotation of z′ about z is uniform due to the symmetry imposed by the laser polarization.

### C. Time-dependent non-adiabatic alignment simulations

The theory of non-adiabatic alignment of asymmetric top molecules by intense laser pulses has been studied in previous publications.^27,28^ In this section, we briefly describe how that theory is used to calculate the angular distribution and degree of alignment of 4-fluorobenzotrifluoride after interaction with a linearly polarized laser pulse. The rotational wavefunction of an asymmetric top molecule can be expanded in the time independent asymmetric top basis set,

$$\left|\left.\Psi_{J_{i}\tau_{i}M_{i}}\left(t\right)\right\rangle \right.=\sum_{J\tau M}B_{J_{i}\tau_{i}M_{i}}^{J\tau M}\left(t\right)\left|\left.J\tau M\right\rangle \right.,$$ (10)

where $J_{i}$, $\tau_{i}$, $M_{i}$, and J, τ, M are the asymmetric top quantum numbers for the initial states and excited states, respectively. To calculate the numerical coefficients $B_{J_{i}\tau_{i}M_{i}}^{J\tau M}\left(t\right)$, it is convenient to expand the asymmetric top basis set $\left|\left.J\tau M\right\rangle \right.$ in the symmetric top basis set $\left|\left.\mathrm{JKM}\right\rangle \right.$,

$$\left|\left.J\tau M\right\rangle \right.=\sum_{K}a_{\tau }^{\mathrm{JKM}}\left|\left.\mathrm{JKM}\right\rangle \right.,$$ (11)

where $a_{\tau }^{\mathrm{JKM}}$ can be obtained by solving the eigenvalue problem,^29^

$$H_{\mathrm{rot}}\left|\left.J\tau M\right\rangle \right.=E^{J\tau M}\left|\left.J\tau M\right\rangle \right.,$$ (12)

where $E^{J\tau M}$ is the energy associated with the state $\left|\left.J\tau M\right\rangle \right.$, and $H_{\mathrm{rot}}$ is the rotational Hamiltonian given by

$$H_{\mathrm{rot}}=\frac{J_{a}^{2}}{2I_{\mathrm{aa}}}+\frac{J_{b}^{2}}{2I_{\mathrm{bb}}}+\frac{J_{c}^{2}}{2I_{\mathrm{cc}}}=AJ_{a}^{2}+BJ_{b}^{2}+CJ_{c}^{2},$$ (13)

where $I_{\mathrm{aa}}$, $I_{\mathrm{bb}}$, and $I_{\mathrm{cc}}$ are the principal moments of inertia with $I_{\mathrm{aa}}\leq I_{\mathrm{bb}}\leq I_{\mathrm{cc}}$.

From Eqs. (10) and (11),

$$\left|\left.\Psi_{J_{i}\tau_{i}M_{i}}\left(t\right)\right\rangle \right.=\sum_{\mathrm{JKM}}C_{J_{i}\tau_{i}M_{i}}^{\mathrm{JKM}}\left(t\right)\left|\left.\mathrm{JKM}\right\rangle \right.,$$ (14)

where

$$C_{J_{i}\tau_{i}M_{i}}^{\mathrm{JKM}}\left(t\right)=\sum_{\tau }B_{J_{i}\tau_{i}M_{i}}^{J\tau M}\left(t\right)a_{\tau }^{\mathrm{JKM}}.$$ (15)

Since

$$B_{J_{i}\tau_{i}M_{i}}^{J\tau M}(t=0)=\delta_{J_{i}J}\delta_{\tau_{i}\tau }\delta_{M_{i}M},$$ (16)

the initial condition is

$$C_{J_{i}\tau_{i}M_{i}}^{\mathrm{JKM}}\left(t=0\right)=a_{\tau_{i}}^{J_{i}KM_{i}}.$$ (17)

The coefficients $C_{J_{i}\tau_{i}M_{i}}^{\mathrm{JKM}}\left(t\right)$ can be determined by solving the time-dependent Schrödinger equation,

$$i\hbar \frac{\partial \left|\left.\Psi_{J_{i}\tau_{i}M_{i}}\left(t\right)\right\rangle \right.}{\partial t}=H(t)\left|\left.\Psi_{J_{i}\tau_{i}M_{i}}\left(t\right)\right\rangle \right..$$ (18)

Within the rigid rotor approximation, the Hamiltonian is given by

$$H\left(t\right)\approx H_{\mathrm{rot}}+H_{\mathrm{ind}}.$$ (19)

Since fluorobenzotrifluoride is an orthorhombic molecule, the induced interaction Hamiltonian can be represented as follows:^30^

$$\begin{array}{c} H_{\mathrm{ind}}=-\frac{\varepsilon^{2}\left(t\right)}{4}\left[\alpha^{\mathrm{ZX}}{\text{cos}}^{2}\gamma +\alpha^{\mathrm{YX}}{\text{sin}}^{2}\gamma {\text{ sin}}^{2}\xi \right]\\ =-\frac{\varepsilon^{2}\left(t\right)}{4}\left\{\frac{\alpha^{\mathrm{ZX}}+\alpha^{\mathrm{ZY}}}{3}D_{00}^{2}-\frac{\alpha^{\mathrm{YX}}}{\sqrt{6}}\left[D_{02}^{2}+D_{0-2}^{2}\right]\right\}, \end{array}$$ (20)

where $\varepsilon \left(t\right)$ is an envelope function describing the shape of the laser pulse, and $\alpha^{\mathrm{ZX}}=\alpha_{\mathrm{ZZ}}-\alpha_{\mathrm{XX}},\;\alpha^{\mathrm{YX}}=\alpha_{\mathrm{YY}}-\alpha_{\mathrm{XX}}$, $\alpha^{\mathrm{ZY}}=\alpha_{\mathrm{ZZ}}-\alpha_{\mathrm{YY}}$ are generalized polarizability anisotropies with $\alpha_{\mathrm{kk}}$ being the body-fixed components of the polarizability tensor. A, B, and C are rotational constants for a given molecule, and $\left(\phi ,\gamma ,\xi \right)$ are Euler angles. $D_{\mathrm{qs}}^{2}$ are Wigner matrices and follow the notation of Zare,^29^

$$\left\langle \mathrm{jkm}|D_{\mathrm{qs}}^{2}|j\prime k\prime m\prime \right\rangle ={\left(-1\right)}^{k^{\prime }+m^{\prime }}\sqrt{\left(2j+1\right)\left(2j\prime +1\right)}\left(\begin{array}{cc} j & \begin{array}{cc} 2 & j\prime \end{array}\\ m & \begin{array}{cc} q & -m\prime \end{array} \end{array}\right)\left(\begin{array}{cc} j & \begin{array}{cc} 2 & j\prime \end{array}\\ k & \begin{array}{cc} s & -k\prime \end{array} \end{array}\right),$$ (21)

where the selection rules $\left|j-2\right|\leq j\prime \leq j+2$, k′=k, k±2, follow from the properties of the 3 − j symbol.

Symmetry in ϕ, imposed by the linearly polarized laser field, constrains M such that $M=M_{i}$. Then, Eq. (14) becomes

$$\begin{array}{c} \left|\left.\Psi_{J_{i}\tau_{i}M}\left(\phi ,\gamma ,\xi ,t\right)\right\rangle \right.=\sum_{\mathrm{JK}}C_{J_{i}\tau_{i}M}^{\mathrm{JKM}}\left(t\right)\left|\left.\mathrm{JKM}\right\rangle \right.\\ =\sum_{\mathrm{JK}}C_{J_{i}\tau_{i}M}^{\mathrm{JKM}}\left(t\right){\left[\frac{2J+1}{8\pi^{2}}\right]}^{\frac{1}{2}}D_{\mathrm{MK}}^{J*}\left(\phi ,\gamma ,\xi \right). \end{array}$$ (22)

After calculating the coefficients, we simulate the probability density at a given time, *t*, by a weighted sum of excited state wavefunctions,

$$\rho \left(\phi ,\gamma ,\xi ,t\right)=\sum_{J_{i}\tau_{i}M}W_{J_{i}\tau_{i}M}\left(T\right){\left|\Psi_{J_{i}\tau_{i}M}\left(\phi ,\gamma ,\xi ,t\right)\right|}^{2}=\sum_{J_{i}\tau_{i}M}W_{J_{i}\tau_{i}M}\left(T\right)\sum_{J^{\prime }K^{\prime }}\sum_{\mathrm{JK}}C_{J_{i}\tau_{i}M}^{J^{\prime }K^{\prime }*}\left(t\right)C_{J_{i}\tau_{i}M}^{\mathrm{JK}}\left(t\right)\frac{{\left[\left(2J^{\prime }+1\right)\left(2J+1\right)\right]}^{\frac{1}{2}}}{8\pi^{2}}D_{{\mathrm{MK}}^{\prime }}^{J^{\prime }}\left(\phi ,\gamma ,\xi \right)D_{\mathrm{MK}}^{J*}\left(\phi ,\gamma ,\xi \right),$$ (23)

where $\Psi_{J_{i}\tau_{i}M}$ represents the wavefunction with initial state $\left.|J_{i}\tau_{i}M\right\rangle$. The weights are given by the Boltzmann distribution, $W_{J_{i}\tau_{i}M}\left(T\right)$, which is determined by the rotational temperature, *T*, of the molecular system.

The observables of interest for an asymmetric top aligned by a linear laser field are the degrees of alignment in γ and ξ given by $\left\langle {\text{cos}}^{2}\gamma \right\rangle$ and $\left\langle {\text{cos}}^{2}\xi \right\rangle$, with ϕ symmetric. We calculate the degree of alignment in each with

cos2γ=∫∑JiτiMWJiτiMT ΨJiτiMϕ,γ,ξ,t2cos2γ dΩ, (24)

cos2ξ=∫∑JiτiMWJiτiMT ΨJiτiMϕ,γ,ξ,t2cos2ξ dΩ, (25)

where Ω is the solid angle followed by the notation of Zare.^29^

## III. EXPERIMENTAL SETUP

The sample is 98% 4-fluorobenzotrifluoride from Sigma-Aldrich and is used without further purification. The experimental setup has been described in detail previously^15^ and is described only briefly here. The electrons, pump laser pulse, and gas jet intersect at the center of the sample chamber. The electrons diffracted from the sample are captured by a phosphor screen, which is imaged onto the chip of an EMCCD camera. The non-diffracted electrons are collected by a Faraday cup, which serves the dual purpose of eliminating the main beam from the recorded signal as well as measuring the current of the electron beam.

**FIG. 2.**
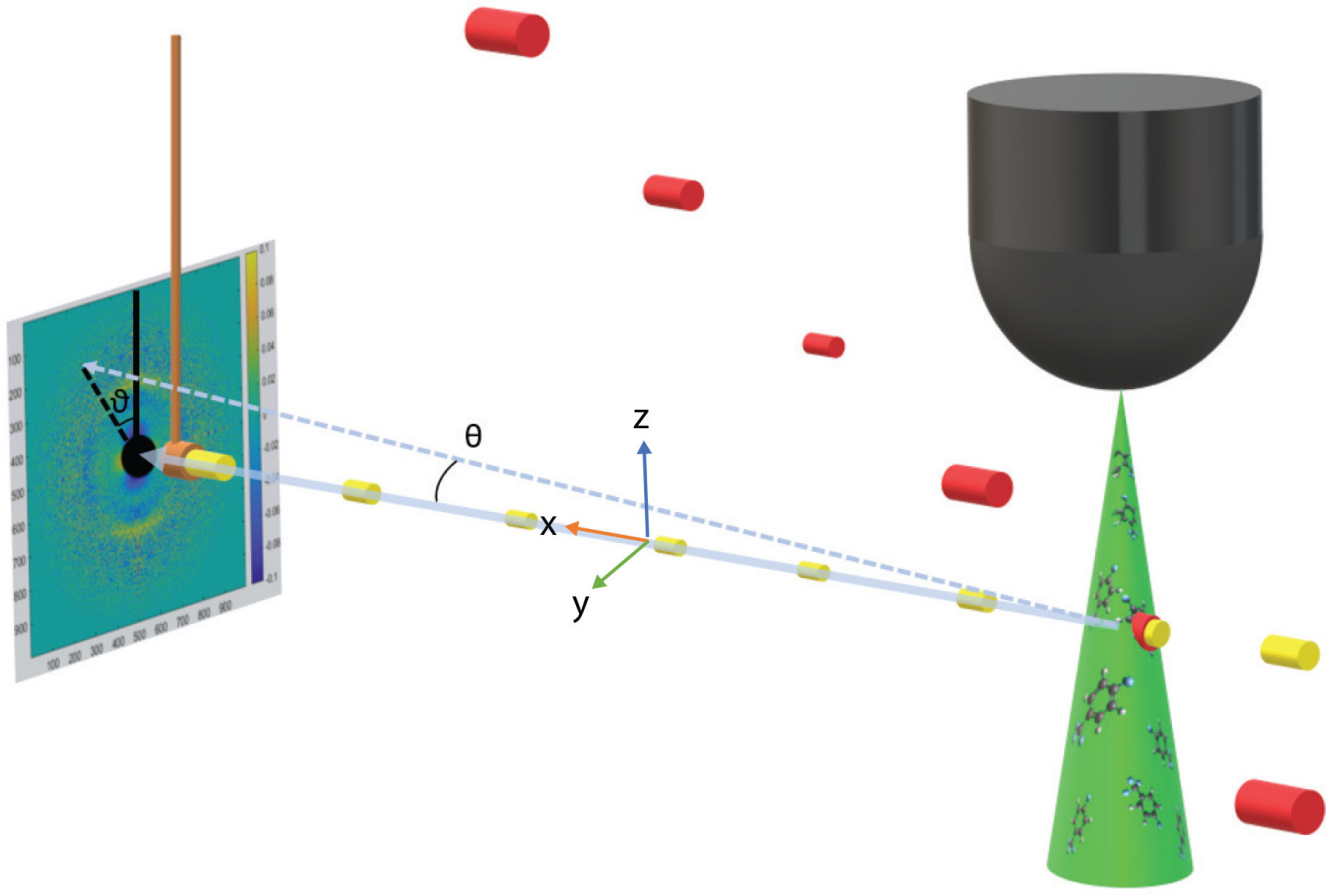
Graphical representation of the experimental setup. Electron beam (yellow cylinders) propagation defines the lab x axis, with vertical being the lab z axis. The laser path (red cylinders) is set to be 60° off the electron beam path in the x–y plane, with a tilted intensity front to match the electrons. The radius of the cylinders representing the laser pulses and electron bunches qualitatively show changes in the radius of the beams from the focusing elements. The temporal focus of the two beams is not shown in the figure. The electrons and the laser intersect at their temporal minima inside the gas jet. The main electron beam is intercepted by a copper beam block. The angle θ is the angle between the main electron beam and a scattered electron. The angle ϑ is the angle from vertical to the pixel on the detector associated with the scattered electron. The diffracted electrons are recorded as a function of pump–probe delay.

A Coherent Ti:Sapphire laser produces a 50 fs pulse, with wavelength centered at 800 nm, 5 kHz repetition rate and 9 W average power. The laser pulse is divided with a non-polarizing beam splitter and propagates along two paths. The first path, using approximately 10% of the total power, passes through two BBO crystals to frequency triple the pulse before imaging the beam onto a photocathode to generate electrons. The remaining power (the pump pulse) is sent to a delay stage to control the timing delay between the electrons and laser pulse. The pump pulse is then reflected off a grating to tilt the intensity front. Tilting the pulse reduces the temporal blurring caused by group velocity mismatch.^31,32^ The laser is introduced to the sample chamber at a 60° angle relative to electron propagation. This matches the velocity of the laser and electron beams along the direction of propagation of the electrons. Figure 2 shows how the intensity front of the electron and laser pulses point parallel to electron propagation, as well as how the pulses from each beam overlap temporally. The tilted pulse is imaged onto the gas jet where it is measured to have a 260 *μ*m (V) × 190 *μ*m (H) FWHM diameter and 200 fs pulse duration. The average power of the laser beam was measured to be 4.92 W just before entering the chamber.

The electron pulses generated from the photocathode are accelerated by a DC electric field to 90 keV and pass through an RF cavity to compress the bunch longitudinally at the sample position. The electron beam is transversely focused at a position between the gas jet and phosphor screen using a pair of magnetic solenoids. This allows the electron beam to be small both on the sample and on the phosphor screen. A small beam on the sample minimizes wasted electrons that would propagate outside the width of the gas jet. The size of the beam block used to block the main beam directly relates to data loss at low scattering angles, as seen in Fig. 2. Ensuring a small beam at the phosphor screen allows for use of a smaller beam block, thus maximizing the acquisition of data at small *s*. A 200-*μ*m platinum aperture is placed 10 cm before the gas jet to ensure a small uniform beam on the sample. The transverse focusing of the electron beam allows for a nearly collimated beam around the sample making the diameter of the beam at the sample approximately 200 *μ*m. Approximately 25 000 electrons per pulse pass through the gas jet, corresponding to a 20 pA current measured at the Faraday cup. The beam block is placed approximately 5 mm in front of the phosphor screen by a thin copper wire. A pulsed RF cavity focuses the beam temporally on the sample.^6,8^ The temporal resolution of the instrument, 240 fs, was determined using the fast rotational dynamics of nitrogen molecules.^15^ The frequency and phase of the RF cavity are controlled by a home-built feedback circuit described in detail in Ref. 15. The RF cavity is pulsed to reduce heat while matching the electron and laser repetition rate. The repetition rate of the laser and electrons are inherently synced from the source laser. The sample is delivered continuously in a supersonic jet to the chamber through a conical nozzle with a backing pressure of 570 Torr. The conical hole is laser drilled into the tip of a rounded end stainless steel tube with an interior and exterior hole size of 30 *μ*m and 90 *μ*m, respectively, and length of approximately 300 *μ*m. The sample consists of fluorobenzotrifluoride molecules in a seed gas of helium. To populate the seed gas with the fluorobenzotrifluoride the He is passed through a long cylinder with liquid sample resting on the bottom. The vapor pressure of the liquid fluorobenzotrifluoride is 35 Torr at 25 °C. The He gas carries the vapor from the sample cylinder into the chamber. The flow is kept constant by a mass flow controller and monitored by the in-chamber pressure.

## IV. RESULTS AND DISCUSSION

We discuss here the experimental results. First, we present the static diffraction taken before the molecules interact with the laser. The *sM*, from the azimuthal average of the diffraction patterns, shows good agreement with theoretical predictions to s = 11 Å^−1^. The *sM* is then sine transformed to give one-dimensional (1D) real space information about the atomic distances with the *PDF*. We then describe and discuss the anisotropy found in the 2D diffraction patterns and how it evolves in time. We compare the experimental data to simulations with differing initial rotational temperatures. Next, we demonstrate experimentally the retrieval of 2D structural information via the Δ*MPDF*. In contrast to the 1D *PDF*, we extract 2D information with distances parallel to and perpendicular to *z*′. Finally, we propose the next step to increase the available information via direct transforms, increasing the alignment with colder molecules. We present simulated Δ*MPDF* of molecules with 1 K initial rotational temperature that show a significant increase in angular resolution of atomic distances.

### A. Static diffraction and pair distribution function

**FIG. 3.**
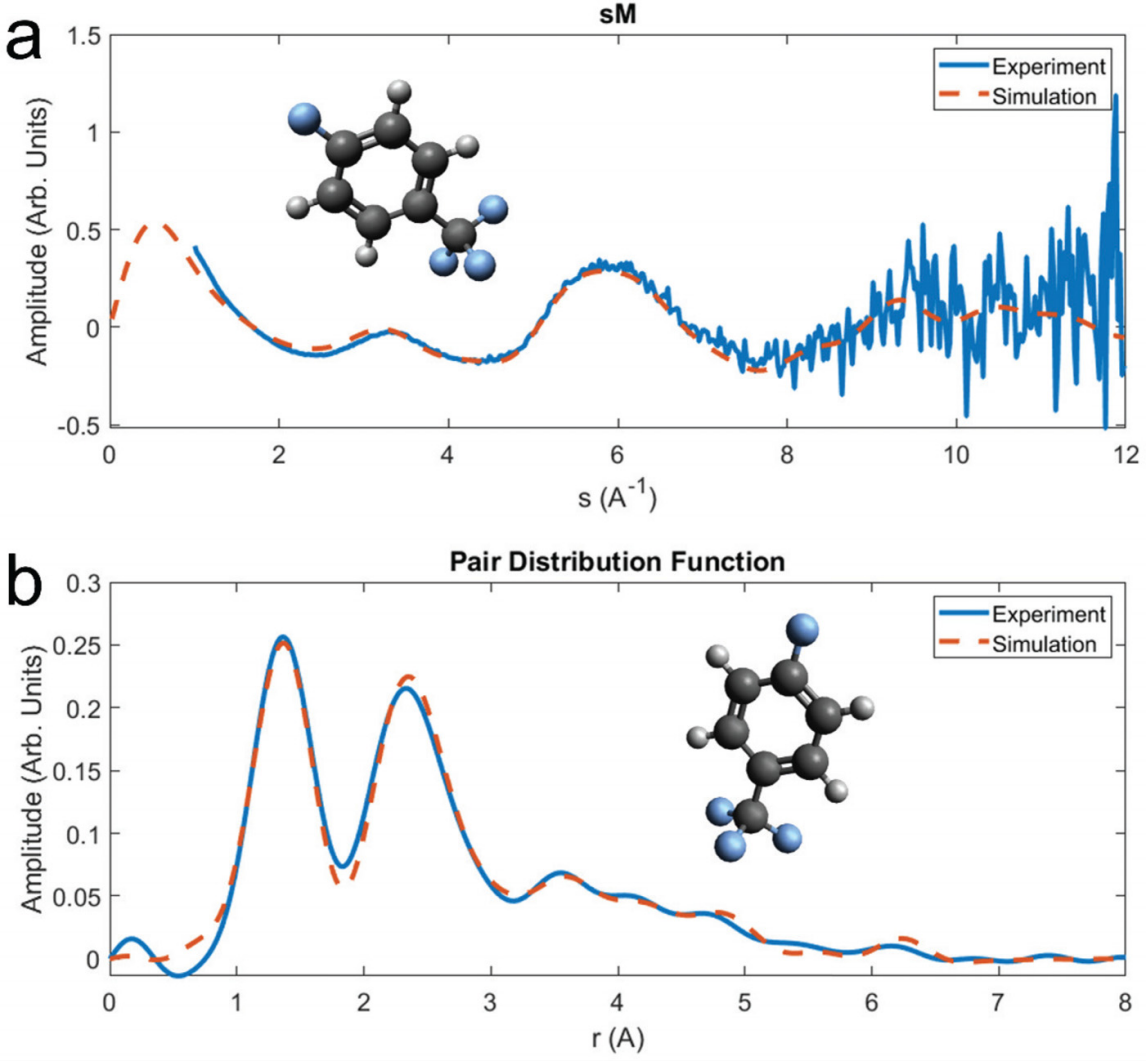
One-dimensional diffraction data and simulations for 4-fluorobenzotrifluoride. (a) Comparison of the sM shows good agreement between simulation and theory for s < 11 Å. (b) Pair distribution function produced from the sM in (a). The low s values for the experimental data are filled with theoretical values prior to the sine transform.

The one-dimensional information is extracted by azimuthally averaging around the center of diffraction. The atomic scattering and background are fit and removed from the total scattering using the zero fitting method.^16^ Figure 3(a) shows the 1D *sM* for the experiment and theoretical simulation. The simulated *sM* is produced by optimizing the structure from previous literature using ORCA software.^33^ Excellent agreement in the 1D signal is seen to almost the edge of the detector at 11 Å^−1^. The increase in noise at higher values of *s* seen in the *sM* is a direct result of the decrease in scattering intensity as a function of scattering angle. The pair distribution function (PDF) is shown in Fig. 3(b). The *PDF* is produced from a sine transform of the *sM*. Peaks in the *PDF* correspond to interatomic distances found in the molecule. To remove artifacts from the transform, low s data that are missing due to the beam stop is filled in with theoretical values. Agreement in the *PDF*, both in position and amplitude of the peaks, is seen up to the longest interatomic distance. Due to the random orientation of the molecules in the gas jet only 1D information is extractable here, with many of the interatomic distances overlapping. The ability to resolve individual distances is limited by $s_{\text{max}}$, which is determined by the signal to noise and the area of the detector with the resolution in real space being approximately $\frac{2\pi }{s_{\text{max}}}$. However, if angular resolution is introduced, distances that overlap in the *PDF* can be resolved if the bond angles are different.

### B. Time-dependent anisotropy

The linearly polarized laser pulse induces excited rotational states in the molecule, as detailed above. The evolution of the molecular ensemble manifests as anisotropy in the diffraction patterns. The anisotropy is quantified by comparing conical sections of $I(\vec{s},t)$ parallel (V) and perpendicular (H) to the laser polarization. The sections cover from the edge of the beam block at s≈1.1 Å to s = 2 Å with an opening angle of $\pm \frac{\pi }{10}$ radians. The anisotropy is monitored as a function of time by a normalized difference of the two sections given by

$$\mathrm{Anisotropy}\left(t\right)=\frac{H-V}{H+V}.$$ (26)

Simulated anisotropy curves are calculated by applying the same process to simulated diffraction data. A diffraction signal is calculated using a weighted average over molecular orientations using Eq. (1), where $\vec{r}=[r_{x}\hat{x},\;r_{y}\hat{y},\;r_{z}\hat{z}]$ and $\vec{s}=k[(\text{cos}\left(\theta \right)-1)\hat{x},\;\text{sin}\left(\theta \right)\text{cos}\left(\vartheta \right)\hat{y},\text{sin}\left(\theta \right)\text{sin}\left(\vartheta \right)\hat{z}]$. We use a sampling rate of 41 for γ between the values of 0 and π, 80 for *ξ* between 0 and 2π, and 80·sin(γ) for ϕ between 0 and 2π. In total, 164 480 diffraction patterns are produced and combined based on the simulated time-dependent angular distributions.

The angular distributions are calculated as described in Sec. II C using the measured laser parameters. Calculating the coefficients $C_{J_{i}\tau_{i}M_{i}}^{\mathrm{JKM}}\left(t\right)$ is the most computationally expensive part of retrieving the angular distributions. Equation (12) is solved numerically using a built in MATLAB function ode45 based on the Runge–Kutta (4,5) method. States with a weight below 1% of the weight of the highest populated state are ignored during the calculations. Simulations for initial states with temperatures T = 1, 10, and 50 K take 2.5, 154.9, and 4538.2 core-hours, respectively. The actual time to calculate the coefficients is reduced by separating the tasks and using parallel computing. Computing jobs are separated to run on nodes of eight cores each using the Crane cluster at Holland Computing Center. Temperatures of 1 and 10 K are split into two jobs (16 cores) and take 0.4 h (0.06 GB RAM) and 8 h (5.89 GB RAM), respectively. The 50 K temperature simulations utilize nine nodes (72 cores) and ran for 67 h (181.7 GB RAM). Calculations at higher rotational temperatures are currently not practical with our computing resources.

**FIG. 4.**
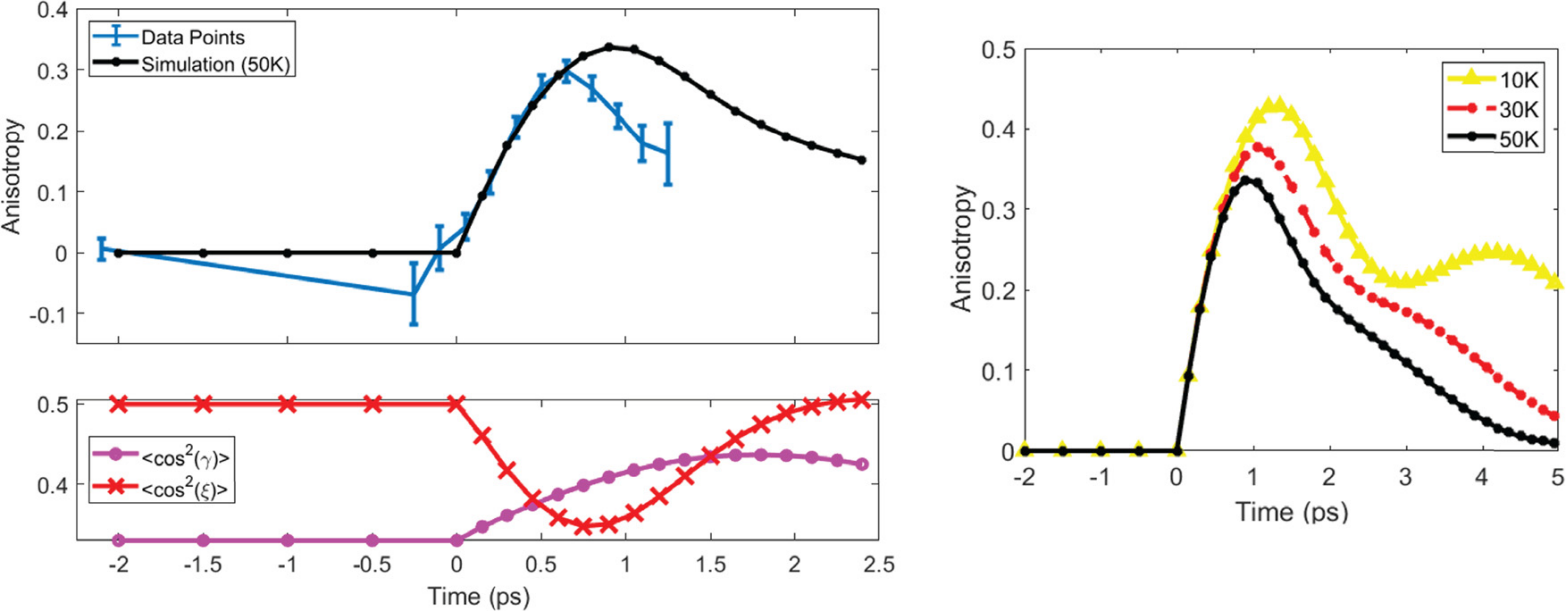
(a) Anisotropy signal measured as a function of time for experimental data (blue, error bars) and molecules simulated with 50 K rotational temperature (black, circles). (b) The ⟨cos^2^⟩ values for the two most polarizable axes of the molecule, γ (magenta, circle) and ξ (red, x) at 50 K. (c) The simulated anisotropy signal over the first 5 ps for three rotational temperatures 50 K (black, circle), 30 K (red, dashed-circle), and 10 K (yellow, triangle).

Figure 4(a) shows the experimental (blue with error bars) and simulated (black, circles) anisotropy signal generated from Eq. (21). The simulated values were produced using a 50 K initial rotational temperature. The experimental data and the simulations both exhibit a quick rise and slow decay. The fast-rising signal is due to a quick anti-alignment in the second most polarizable axis, ξ [Fig. 4(b) red, x]. This is combined with a slower alignment in γ [Fig. 4(b) magenta, circle] producing a shoulder at later times. Due to the heavy computational load that comes with increasing temperature we are limited to 50 K as our maximum simulated temperature. The amplitude of the anisotropy is determined in part by the spatial overlap in the interaction region. This requires normalization of the signal to properly compare with the simulations. The amplitude of the experimental signal relative to the simulations is set by examining the trend seen in Fig. 4(c) for different initial rotational temperatures. The width and amplitude of the anisotropy signal both decrease with increasing temperature, while the rising edge remains constant. The time zero in Fig. 4(a) is set by overlapping the rising edge of the experiment and simulation. We estimate our initial rotational temperature to be between 60 and 100 K based on qualitative examination of the trends. The experimental anisotropy values are increased by a factor of 8 to match the width and amplitude of the data with the trend seen in the simulations. This gives a volume-averaged interaction percentage of 12.5%. This percentage is a combination of the spatial overlap between the electron beam and the laser pulse, and the intensity gradient over the Gaussian laser beam.

### C. Retrieval of 2D structural information

**FIG. 5.**
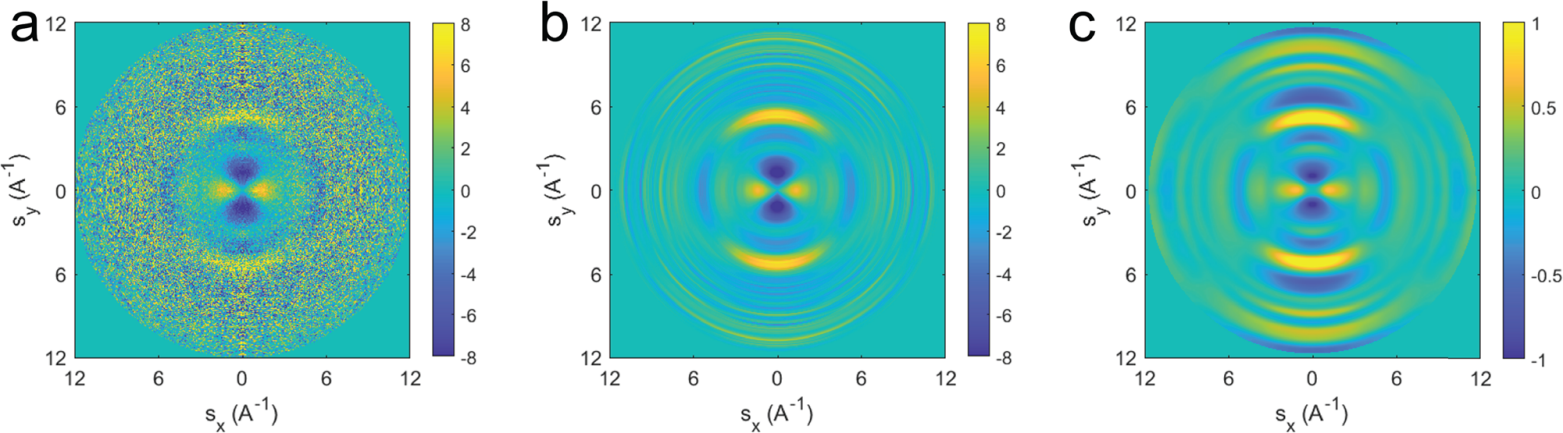
Diffraction difference images for (a) the experimental data before Legendre projection, (b) experimental data built from Legendre orders 2 and 4, and (c) simulated data. Both (b) and (c) have data smoothly interpolated to zero at the center from the surrounding values at 1.1 Å.

Figure 5 shows the 2D Δ*sM* at the time of peak anisotropy. The experimental Δ*sM* after standard noise reduction techniques is seen in Fig. 5(a) (see the Appendix for data processing). The missing region at the center of the pattern is filled in by smooth interpolation to zero at the origin; we do not use theoretical values to avoid biasing the structure retrieval. The pattern in Fig. 5(a) is projected onto the Legendre polynomials to produce the diffraction pattern seen in Fig. 5(b). Figure 5(b) shows good agreement with the simulated data in Fig. 5(c) nearly to the edge of the image. The ability to match experimental signal to simulated values up to 11 Å^−1^ in 2D gas-phase diffraction-difference images is approximately a factor of 2 improvement over previous experiments^22^ and paves the way for full structural retrieval methods to be applied with a moderately higher level of alignment.^24^

**FIG. 6.**
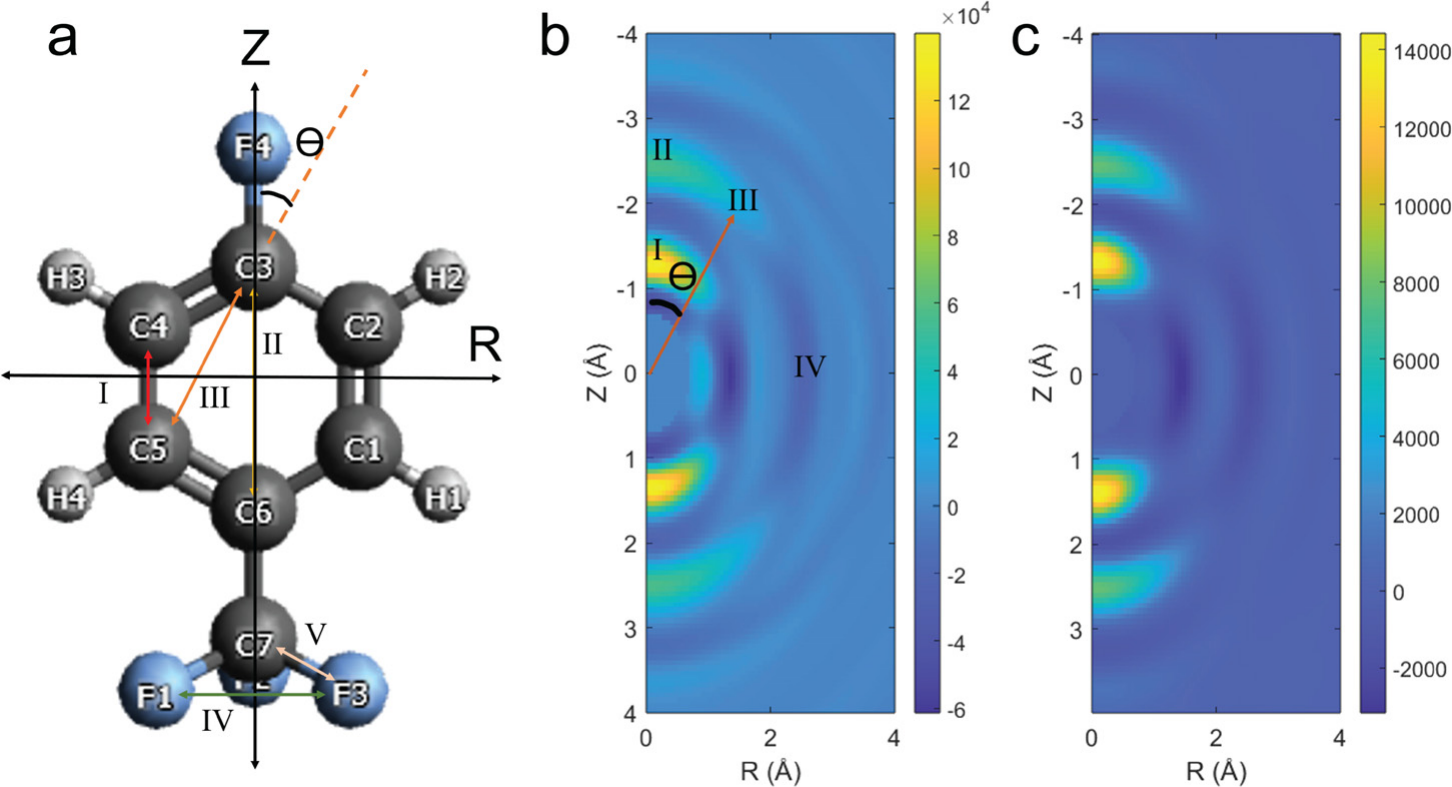
(a) Theoretical structure for 4-fluorobenzotriflouride with atoms numbered and labeled by atomic symbol. The numbered arrows indicate distances that correspond to peaks found in (b) and (c). (b) and (c) The Δ*MPDF* from the transformed (b) experimental and (c) simulated Δ*sM* at the peak of anisotropy. The numbers found in (b) represent where peaks should be found based on theoretical atomic distances.

Transforming the diffraction patterns into the Δ*MPDF* allows for direct extraction of 2D structural information (distances and angles) in the molecule. A model of the molecule, with atomic labeling by atom type and number, is shown in Fig. 6(a). Cylindrical coordinates are represented with Z and R, parallel and perpendicular to the laser polarization, respectively. Figures 6(b) and 6(c) is the Δ*MPDF*, containing information about the molecular structure expressed in cylindrical coordinates. Due to the symmetry imposed by the laser the Δ*MPDF* are symmetric about Z = 0 (the molecule has no up–down preference). Several distances are highlighted in both the molecular model and the Δ*MPDF*. Distances aligned with either the Z axis [distances I, C4–C5 and C1–C2 at (Z,R) = (1.38 Å, 0 Å), and II, C3–C6 at (Z,R) = (2.75 Å, 0 Å)] or R axis [distance IV, F1–F3, F1–F2, and F2–F3 at (Z,R) = (0 Å, 2.18 Å) and C4–C2 and C5–C1 at (Z,R) = (0 Å, 2.41 Å)] are separated in the Δ*MPDF*. Arrows in Fig. 6(a) are placed at only one atomic distance, even when several distances contribute to a peak in the Δ*MPDF*, for visual clarity. This is in contrast to the 1D PDF [Fig. 3(b)] where all the distances from 2 to 3 Å are contained within a single peak. This clearly demonstrates experimentally, for the first time, the capability to extract 2D structural information from diffraction of aligned asymmetric top molecules.

### D. Simulations of molecules at rotational temperature of 1 K

The limited level of alignment in the experiment means some distances and angles, such as II and III, cannot be resolved with the direct transform. Improving the alignment will help to separate the atomic distances blurred together through the angular distribution of the molecular ensemble. One method of increasing the alignment is to lower the initial rotational temperature. We simulate the structure retrieval for the case of an initial rotational temperature of 1 K, which can be reached using seeded gas jets and skimmers.

**FIG. 7.**
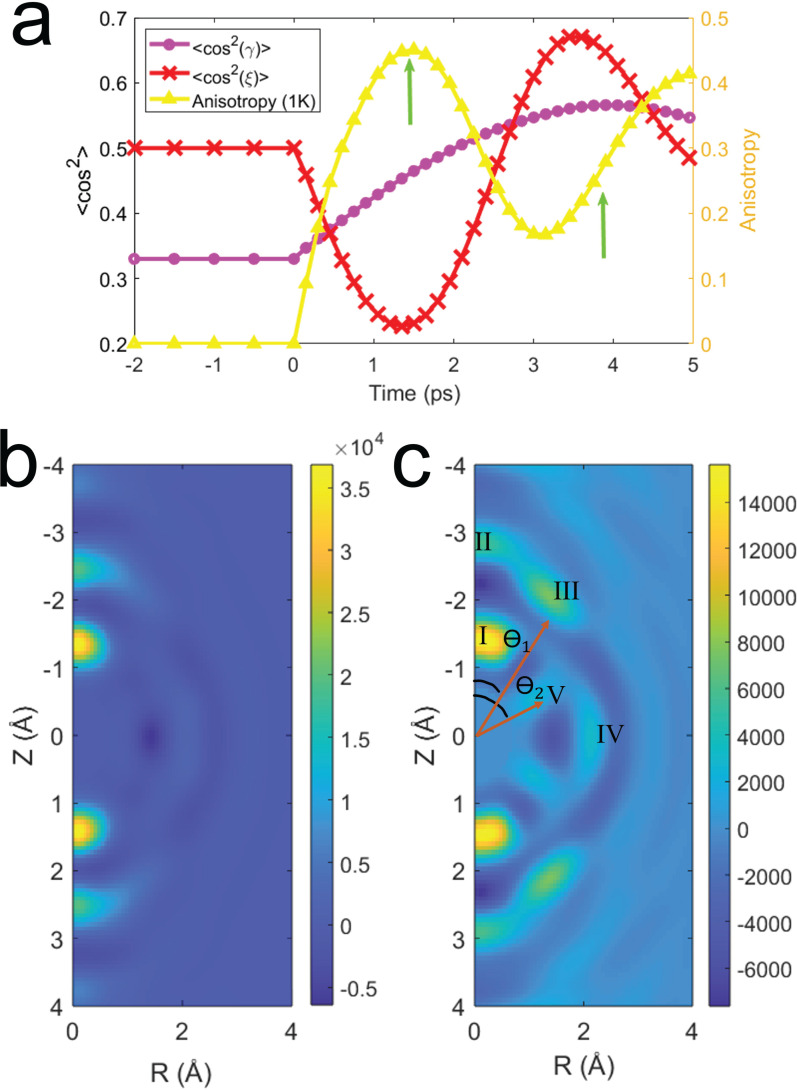
(a) Left axis: Observables of alignment, ⟨cos^2^( γ)⟩ (magenta, circle) and ⟨cos^2^(ξ)⟩ (red, x). Right Axis: Anisotropy in the simulated ΔI for 1 K initial rotational temperature. The Δ*MPDF* for the (b) peak of anisotropy and (c) γ peak for simulated 1 K diffraction difference patterns.

The ⟨cos^2^( γ)⟩ (magenta, circle) and ⟨cos^2^(ξ)⟩ (red, x) for a molecular ensemble with initial rotational temperature 1 K are shown in Fig. 7(a), along with the anisotropy (yellow, triangle) calculated from the simulated diffraction patterns. At this temperature, the dynamics in γ and ξ are drastically different and the separation of the alignment peak in γ and anti-alignment peak in ξ allow for easier understanding of how they affect the anisotropy. It is clear from Fig. 7(a) that anti-alignment in ξ causes a strong anisotropy signal, and alignment in ξ causes the anisotropy signal to drop. A more careful interpretation of the plots in Fig. 7(a) also shows a positive anisotropic signal with alignment in γ. The green arrows in Fig. 7(a) are at two points of interest in the evolution of the molecular ensemble. Figures 7(b) and 7(c) show the Δ*MPDF* for these two points. The first point of interest is the peak of anisotropy at t = 1.5 ps [Fig. 7(b)] and the second is the peak of alignment in γ at t = 3.9 ps [Fig. 7(c)].

Examination of the simulated Δ*MPDFs* at T = 1 K shows that taking data at the peak of alignment in γ has significant advantage over taking data at the peak of anisotropy. This advantage does not exist in the simulations with a temperature of 50 K as the Δ*MPDFs* are nearly indistinguishable at the same points of interest. While Fig. 7(b) shows significant improvement over Figs. 6(b) and 6(c), with a sharp angle seen between II and III, there is no clear separation between the distances. In Fig. 7(c), a clear separation of II and III is evident, allowing for extraction of the distance and angle of the off-axis atomic distances. Highlighted atomic distance III, between C3–C5, has a distance and angle ( ${ϴ}_{1})$ of 2.53 Å and 34°, respectively. These values are in close agreement with the theoretical values of 2.38 Å and 31°. Similarly for V, the distance and angle ( ${ϴ}_{2}$) for the C7–F1, F2, F3 umbrella, not seen in the simulations at T = 50 K, has extracted values of 1.28 Å and 65° closely agreeing with theoretical values of 1.36 Å and 68°. This is a significant improvement in directly accessible structural information compared to the 1D case, where the five distances discussed above are combined into two peaks centered at 1.36 and 2.35 Å. The application of the transforms at T = 1 K allow for recovery of 2D information directly from the diffraction patterns. The use of phase retrieval or structural refinement algorithms might result in more accurate structural parameters; however, the direct 2DFT-A transform is sufficient to extract information well beyond what is accessible with the 1D PDF without any *a priori* structural information.

Recent work using a truncated adiabatic pulse has shown a high degree of field free alignment of large molecules in helium nanodroplets^34^ and three-dimensional field-free alignment of generic asymmetric-top molecules.^35^ The high degree of alignment would allow for better angular resolution in the Δ*MPDF,* and the long alignment window could allow for imaging of structural changes of a molecule after photo-excitation.

## V. CONCLUSION

We have shown the direct extraction of 2D structural information, distances, and angles, from gas-phase UED of the complex molecule 4-fluorobenzotrifluoride. We resolve distances both parallel to and perpendicular to the laser polarization axis from simple transformations of experimental diffraction images, without any *a priori* knowledge of the target molecular structure. This was enabled by recording the diffraction pattern at the peak of the laser-induced alignment and capturing a momentum transfer range that was two times higher than previous experiments with aligned asymmetric tops. We use a direct transform to retrieve the structural information, which shows close agreement with simulations. We further show, by simulation, that reducing the initial temperature to 1 K improves the alignment enough to resolve multiple angles from distances that overlap at the current level of alignment.

## Data Availability

The data that support the findings of this study are available from the corresponding authors upon reasonable request.

## APPENDIX: PROCESSING OF EXPERIMENTAL IMAGES

The images are recorded over several scans with multiple images per scan. Each image is taken with an 8 s integration time and indexed according to stage position. The scans cover the same nominal time points with the stage positions changed to capture the rise and peak of anisotropy. A background image is saved without gas before and after the scans. A mask is used to block any pixels affected by the beam block. A threshold is set near the saturation limit of the camera. Any pixels with counts above the threshold are deemed “hot-pixels” and are ignored. The background is then subtracted from each experimental image. The images in each scan are averaged based on stage position. Radial outliers are then removed from images by first determining the average and standard deviation as a function of scattering angle. Pixels with amplitude over four standard deviations away from the average are ignored. Images are then normalized by the total counts in the region between 3.7 and 7.5 Å^−1^. The normalized images are first projected onto the Legendre polynomials to verify the assumed symmetries. The normalized images are then quarter averaged (QA) to improve the statistics for each pixel. QA consists of separating each quadrant of the image and averaging them together based on the symmetry found in the patterns. Following QA, the anisotropy as a function of stage position is again calculated for each scan. Images are then time-indexed (ti) based on their temporal distance from the peak of anisotropy within each scan. Images with identical ti are combined. Images at the peak of anisotropy, and one ti before and after the peak, are combined to improve the signal to noise. The Δ*sM* is then generated by subtracting the combined static QA image from the combined QA image at the peak of anisotropy. The combined Δ*sM* images have missing low scattering angle values due to the beam block. The *sM*, by definition, is zero at *s* = 0 and thus the images can be filled smoothly by interpolation from the values at ∼1.1 Å^−1^ to zero at the center, avoiding the use of theory as in the 1D case. The filled Δ*sM* images are then projected onto the Legendre polynomials and, due to the symmetry imposed by the laser, only even orders contribute. Orders above four have negligible amplitude, and thus, only the second and fourth orders are kept. Projections of the simulations confirm the contribution of higher orders is negligible. The images are then rebuilt from the projections into diffraction patterns similar to that seen in Fig. 5(b). The patterns are then transformed into the Δ*MPDF* according to Sec. II A.
